# Association of Recreational Cannabis Legalization With Alcohol Use Among Adults in the US, 2010 to 2019

**DOI:** 10.1001/jamahealthforum.2022.4069

**Published:** 2022-11-18

**Authors:** Vandana Macha, Rahi Abouk, Coleman Drake

**Affiliations:** 1 Department of Economics, University of Pittsburgh, Pittsburgh, Pennsylvania; 2 Department of Economics, Finance, and Global Business, William Patterson University, Wayne, New Jersey; 3 Department of Health Policy and Management, University of Pittsburgh School of Public Health, Pittsburgh, Pennsylvania

## Abstract

**Importance:**

In the US, cannabis use has nearly doubled during the past decade, in part because states have implemented recreational cannabis laws (RCLs). However, it is unclear how legalization of adult-use cannabis may affect alcohol consumption.

**Objective:**

To estimate the association between implementation of state RCLs and alcohol use among adults in the US.

**Design, Settings, and Participants:**

This was a cross-sectional study of 4.2 million individuals who responded to the Behavioral Risk Factor Surveillance System in 2010 to 2019. A difference-in-differences approach with demographic and policy controls was used to estimate the association between RCLs and alcohol use, overall and by age, sex, race and ethnicity, and educational level. Data analyses were performed from June 2021 to March 2022.

**Exposures:**

States with RCLs, as reported by the RAND−University of Southern California Schaeffer Opioid Policy Tools and Information Center.

**Main Outcomes and Measures:**

Past-month alcohol use, binge drinking, and heavy drinking.

**Results:**

Of 4.2 million respondents (median age group, 50-64 years; 2 476 984 [51.7%] women; 2 978 467 [58.3%] non-Hispanic White individuals) in 2010 through 2019, 321 921 individuals lived in state-years with recreational cannabis laws. Recreational cannabis laws were associated with a 0.9 percentage point (95% CI, 0.1-1.7; *P* = .02) increase in any alcohol drinking but were not significantly associated with binge or heavy drinking. Increases in any alcohol use were primarily among younger adults (18-24 years) and men, as well as among non-Hispanic White respondents and those without any college education. A 1.4 percentage point increase (95% CI, 0.4-2.3; *P* = .006) in binge drinking was also observed among men, although this association diminished over time.

**Conclusions and Relevance:**

This cross-sectional study and difference-in-differences analysis found that recreational cannabis laws in the US may be associated with increased alcohol use, primarily among younger adults and men.

## Key Points

**Question:**

How do recreational cannabis laws affect alcohol use among adults in the US?

**Findings:**

In this cross-sectional study with a difference-in-differences analysis of 4.2 million adults in all 50 states from 2010 to 2019, recreational cannabis laws were associated with a 0.9 percentage point increase in any alcohol use among the population overall but not in binge or heavy drinking. Results were primarily determined by younger adults (18-24 years) and men.

**Meaning:**

These findings suggest that increased alcohol use may be an unintended consequence of recreational cannabis laws.

## Teaser

This cross-sectional study and difference-in-differences analysis examines the association of state-level recreational cannabis laws with increased alcohol use among adults in these states.

## Introduction

Alcohol and cannabis are the first and third most-used substances in the US.^1^ Past-month adult cannabis use has nearly doubled during the past decade, increasing from 6.9% in 2010 to 11.9% in 2019.^2^ During each year from 2011 to 2015, there were approximately 95 000 alcohol-attributable deaths in the US.^3,4^

Cannabis use may contribute to poor health outcomes in adults, both by itself and when used with alcohol. By itself, cannabis use increases the risk of cannabis use disorder, nausea, and hyperemesis, and may increase the risk of some mental health conditions.^5^ Co-use of cannabis and alcohol may increase the probability of unsafe driving, beyond the risk associated with consuming either substance alone.^6,7^ In addition, co-use also has been associated with increased impulsivity, which may give rise to potentially dangerous behaviors.^8^ In addition, simultaneous use of cannabis and alcohol has been associated with increased alcohol use frequency and quantity and an increased risk of drunk driving.^5,8,9,10,11^

Cannabis use has increased alongside widespread liberalization of state cannabis policy.^11,12^ As of August 2022, 38 states had implemented medical cannabis laws, 19 of which also legalized recreational cannabis for adult use.^12^ Recreational cannabis also is legal in the District of Columbia. These recreational cannabis laws (RCLs) have made cannabis accessible to approximately half of adults in the US.^13,14^ They have resulted in a 25% increase in cannabis use by adults.^15^ Although previous studies indicate the percentage increases in cannabis use in response to RCLs have been as large or larger among older adults compared with younger ones, absolute increases in cannabis use have been larger among younger adults.^15,16^ Part of the rationale for these policies is that cannabis is used to treat long-term health conditions, including chronic pain, glaucoma, epilepsy, and anxiety.^17^ There is some evidence that cannabis may be a substitute for opioids in the treatment of chronic pain.^18,19,20^

Previous studies investigating the relationship between cannabis and alcohol use have found mixed evidence.^21,22,23,24^ The findings of 2 studies that used repeated cross-sectional data on college students suggest that alcohol and cannabis are economic *complements*—that demand for one substance increases along with the other.^21,22^ However, the results of a more recent study that used a regression discontinuity design suggest that cannabis and alcohol are *substitutes*—that demand forone substance decreases as demand for the other increases. Specifically, this study found an increase in alcohol consumption and a decline in cannabis use at the legal drinking age of 21 years.^23^ However, another study using the same methods with data from the 1997 National Longitudinal Survey of Youth found no relationship between cannabis and alcohol.^24^

The association between cannabis policies and adult alcohol use also are unclear.^25^ Two studies have found that medical cannabis laws reduced alcohol use,^26,27^ suggesting that cannabis and alcohol are substitutes. However, 2 other studies found that medical laws increased alcohol use, indicating that the 2 substances were economic complements.^16,28^ In particular, little is known about how RCLs affect alcohol use. A recent study found that RCLs increased alcohol use^16^; another study found an increase in co-use of cannabis and alcohol and a decline in the use of alcohol alone.^29^ Both studies used data from the National Survey on Drug Use and Health, which has a sample size of approximately 1-sixth of the size of another publicly available data source, the Behavioral Risk Factor Surveillance System (BRFSS).^30,31^ With 1 exception,^16^ these studies also did not examine the robustness of their findings to bias from staggered implementation, which may significantly bias difference-in-differences (DiD) study designs.^32^

We examined the association between RCLs and alcohol use among adults in the US from 2010 through 2019 using survey data of more than 4.2 million respondents. During the study period, 10 states and the District of Columbia legalized adult-use cannabis. We also evaluated the heterogeneous association of RCLs with alcohol use by age, sex, race and ethnicity, and education level.

## Methods

This cross-sectional study was exempt from review because it used only secondary deidentified data; informed consent was also not required. This study followed Strengthening the Reporting of Observational Studies in Epidemiology (STROBE) reporting guidelines.

We used a DiD approach to estimate the association between RCLs and alcohol use in all 50 states and the District of Columbia from 2010 to 2019. We examined overall associations and subgroup associations by age, sex, race and ethnicity, and education level.

### Data Source

The primary data source for this study was the BRFSS, an annual, repeated cross-sectional survey conducted by the Centers for Disease Control and Prevention in conjunction with the states that collect information on adult health behaviors and outcomes.^30^ We used 2010 to 2019 BRFSS data for information on alcohol use and demographic information. We also used data from the RAND Opioid Policy Tools and Information Center on the implementation dates of cannabis laws, and information on beer and tobacco taxes from the US National Institute on Drug Abuse and the Centers for Disease Control and Prevention.^33,34,35^

### Measurement of Drinking Behavior

Alcohol use was measured and reported per the BRFSS alcohol module definitions. Three survey questions measured any alcohol use, binge drinking, and heavy drinking, all within the past month. Any drinking was defined as having had “at least 1 drink of any alcoholic beverage” in the past month.^36^ Binge drinking was defined as consuming 5 or more drinks for men and 4 or more drinks for women on a single occasion during the past month.^36^ Heavy drinking was defined as consuming more than 14 drinks for men and more than 7 drinks for women per week.^36^ We excluded respondents who had not answered all of these questions.

### Treatment Variable

Our treatment variable or primary variable of interest was an indicator for whether a state had implemented RCLs in a state-quarter. If an RCL was implemented by the end of the first month of a quarter for a specific state, we coded persons in that state-quarter as being *treated* by an RCL.

### Independent Variables

Our analyses controlled for sociodemographic characteristics of the respondents and for policies that may affect alcohol and cannabis use. Sociodemographic controls included age in years (18-24, 25-34, 35-49, 50-64, 65-79, ≥80, unknown), sex, race and ethnicity (Hispanic, non-Hispanic Black, non-Hispanic White, other response, or year was 2010 when race and ethnicity were surveyed differently by the BRFSS), education level (≤high school or equivalent, some college or more), marital status, annual income (<$50 000, ≥$50 000, unknown), whether the respondent had children, student status, unemployment status, and where the survey took place (in a metropolitan statistical area, outside of a metropolitan statistical area, or by cell phone). Race and ethnicity were self-identified on the BRFSS survey instrument. Policy controls included indicators for whether a state had implemented a medical cannabis law and opened at least 1 medical cannabis dispensary in a quarter-year, both of which we measured analogously to RCL implementation; state excise taxes on beer, a standard proxy for state restrictions on alcohol sales^34^; and excise taxes on packs of cigarettes, which may affect whether individuals smoke tobacco or cannabis.

### Statistical Analysis

We used a DiD approach to compare changes in alcohol use across states that implemented RCLs (the treatment group) with those that did not (the comparison group) over time. We estimated multivariate linear regressions models of alcohol use for BRFSS respondent (*i*) in the state (*s*) in year-quarter (*t*):

*Y_ist_ = α + βX_ist_ + δRCL_st_ + θ_s_ + τ_t_ + ϵ_ist_*

where *Y_ist_* is an alcohol use-related outcome (ie, any drinking, binge drinking, or heavy drinking) and *RCL_st_* is a binary indicator for whether an RCL was implemented in state *s* and the year-quarter *t*. The quarter in which the RCL was implemented in a state and every subsequent quarter were considered to be *treated* quarters. We included sociodemographic information and policy controls in *X_ist_* to address potential confounders. State (*θ_s_*) and year-quarter (*τ_t_*) fixed effects controlled for time-invariant state characteristics and secular trends in alcohol use, respectively. We specified the binary outcomes as ranging between 0 and 100 so that the estimates could be interpreted as percentage point (pp) changes in alcohol use.

Difference-in-differences estimates were identified under the parallel trends assumption that, absent the implementation of RCLs, alcohol use in states that implemented RCLs would have trended similarly to comparison states. This assumption cannot be tested, but it can be supported by performing a pre-trends test from an event study model of the form:

*Y_ist_ = α + βX_ist_ + Σ*^2^*_j_* _=_ _–3_
*δj RCL_st_* + *θ_s_ + τ_t_ + ϵ_ist_*

Treating the year prior to RCL implementation as the reference period (*j = – 1*), the coefficients on the summation term can be interpreted as changes in alcohol use vs the year prior to RCL implementation. Using the lead terms (*j ≤ –2*), we conducted a pre-trends test to determine whether there were any differential trends in alcohol use among treatment and comparison states prior to RCL implementation. We also used the lag terms (*j ≥ 0*) to estimate the association between RCLs and alcohol use over time.

We performed several robustness checks. First, we performed covariate balance tests by taking the standardized mean difference of the sociodemographic and policy control variables.^37,38,39^ These tests indicated whether there were differences in observable characteristics between the treatment and comparison groups prior to RCL implementation. Second, we estimated stacked DiD models that eliminated bias from improper comparisons of states that implemented RCLs later than those that implemented them earlier, as well as heterogeneous effects of RCLs across states.^32^ Third, we estimated models that only used BRFSS respondents from states that would implement medical cannabis laws during the study period. This approach limited the comparison group to states with more similar cannabis policy environments to treatment states. Fourth, we estimated models where the treatment and comparison groups had similar tobacco taxes. Like the previous robustness check, this approach limited the comparison group to states with more similar tobacco policy environments. Fifth, we estimated regressions that do not include demographic and policy variables other than RCLs.

The unit of analysis was the BRFSS respondent-quarter. Respondents were weighted with standard BRFSS survey weights. To assist with processing time, we collapsed the results to the state-quarter level prior to estimation, taking weighted means of all outcomes and covariates using the previously mentioned survey weights. This approach was equivalent to respondent-quarter level estimation.

We reported standard errors clustered at the state, the unit of treatment. *P* values were 2-tailed, and statistical significance was defined as *P* < .05. Statistical analyses were performed from June 2021 to March 2022 using the R Statistical Package, version 4.1.3 (R Foundation for Statistical Computing).

## Results

### Study Sample

**Figure 1.**
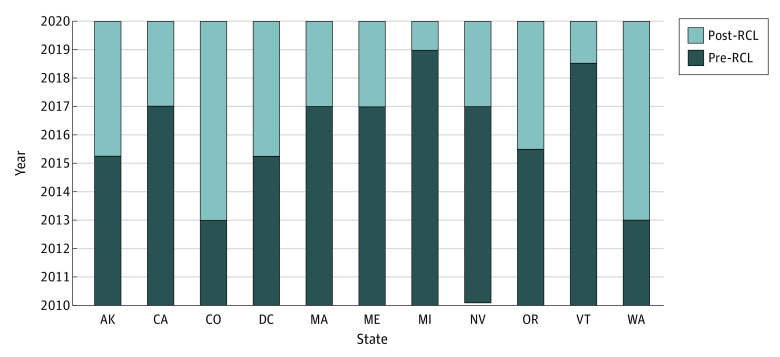
Timing of Recreational Cannabis Laws (RCLs) by Year-Quarter Among Implementing States, 2010 to 2019 States were coded as implementing an RCL in a quarter-year when it was implemented by the end of the first month of that quarter. For example, a state that implemented an RCL by January 31, 2017, was counted as implementing it in the first quarter of 2017. Implementation refers to the date on which the law went into effect, not the date on which it was passed.

Of 4 530 703 total BRFSS respondents in 2010 through 2019, 4 245 824 responded to the questions pertaining to any, binge, and heavy alcohol use and thus were included in the study sample (median age group, 50-64 years; 2 476 984 [51.7%] women; 251 011 [13.3%] Hispanic, 298 594 [10.2%] non-Hispanic Black, 2 978 467 [58.3%] non-Hispanic White, and 717 752 [18.2%] individuals who self-reported being of other race or ethnicity or completed the 2010 survey which used a different approach to collecting these data; Table 1). Of these respondents, 321 921 respondents were in state-years with RCLs (Table 2). Figure 1 shows the states and years in which RCLs were implemented during the study period. Ten states plus the District of Columbia implemented RCLs by 2019 in 6 cohorts specified by year and quarter (Q): (1) Colorado and Washington in 2014Q1; (2) Alaska and the District of Columbia in 2015Q2; (3) Oregon in 2015Q3; (4) California, Maine, Massachusetts, and Nevada in 2017Q1; (5) Vermont in 2018Q3; and (6) Michigan in 2019Q1.

**Table 1.** Baseline Characteristics (Prior to Implementation) of Sample Population in States That Did Not vs Did Implement Recreational Cannabis Laws (RCLs) in 2010 to 2019^a^

Characteristic	RCL implemented		
	No (n = 4 678 372)	Yes (n = 232 447)	Standardized mean difference^b^
**Alcohol drinking behavior (%)**			
Any	48.9	58.2	0.19
Binge	12.0	13.2	0.04
Heavy	5.2	6.6	0.06
**Cannabis and alcohol policies**			
Medical law (%)	28.1	100	2.26
Medical dispensary (%)	10.7	59.5	1.19
Beer tax ($)	0.29	0.30	0.02
Tobacco tax ($)	2.50	3.80	0.58
**Sociodemographic groups**			
Age, y (%)			0.06
18-24	4.9	5.1	
25-34	9.5	9.9	
35-49	19.4	20.0	
50-64	31.5	32.1	
65-79	25.2	24.2	
≥80	8.5	7.4	
Missing	1.1	1.3	
Sex (%)			0.04
Female	59.4	57.3	
Male	40.6	42.7	
Race and ethnicity (%)			0.24
Hispanic	5.5	8.1	
Non-Hispanic Black	7.8	4.6	
Non-Hispanic White	70.8	64.6	
Other^c^	15.9	22.7	
Some college (%)	61.2	68.7	0.16

^a^ Different sets of comparison states were used for each set of states that implemented an RCL in a particular quarter, similarly to the stacked regression models reported in eTable 3 in the Supplement. This "stacked" sample thus has a larger sample size. For example, the comparison states for Oregon, which implemented an RCL in the second quarter of 2015, were all states that did not implement an RCL within 3 years. We determined the means of all treatment states and their respective comparison groups in the years before the treatment states implemented RCLs. We calculated means using standard Behavioral Risk Factor Surveillance System survey weights.

^b^ Covariate imbalance was assessed by estimating standardized mean differences between persons in treatment and comparison states using the approach of Yang and Dalton.^33^ Standard mean differences describe mean differences on the same scale—in terms of SDs—across variables measured on different scales. Imbalances increased with larger standardized means differences. Imbalances with standardized mean differences <0.25 can be addressed by regression adjustment, as we did in this analysis.^34,35^ Medical cannabis laws have standard mean differences that exceed 0.25; therefore, we estimated separate models that limited the sample to states that implemented medical cannabis laws during the study period (additional details are presented in eTable 4 in the Supplement).

^c^ Other includes non-Hispanic respondents who self-identified as American Indian or Alaska Native, Asian, Pacific Islander, or other (other race or ethnicity, did not provide a response, or responded in 2010 when the survey used a different approach to collecting these data).

**Table 2.** Associations of Recreational Cannabis Laws (RCLs) With Alcohol Use Behavior, Overall and by Sociodemographic Groups^a^

Sociodemographic group	Association of RCL with alcohol use [95% CIs] (percentage points)		
	Any drinking	Binge drinking	Heavy drinking
Overall	0.9 [0.1 to 1.7] (0.021)	0.4 [–0.3 to 1] (0.241)	0.3 [–0.1 to 0.6] (0.171)
Age, y			
18-24	3.7 [1.1 to 6.3] (0.006)	1.1 [–0.5 to 2.8] (0.179)	0.1 [–1.2 to 1.4] (0.883)
25-34	1.1 [–0.7 to 3] (0.226)	–0.1 [–1.7 to 1.6] (0.951)	0.8 [–0.2 to 1.7] (0.105)
35-49	1.1 [–0.7 to 2.8] (0.239)	0.7 [–0.2 to 1.6] (0.125)	0.2 [–0.3 to 0.8] (0.398)
50-64	0.3 [–1.1 to 1.6] (0.674)	0.7 [–0.3 to 1.8] (0.177)	0.3 [–0.5 to 1.1] (0.419)
65-79	1.0 [–0.2 to 2.2] (0.104)	–0.2 [–0.7 to 0.3] (0.443)	0 [–0.7 to 0.6] (0.911)
≥80	–2.3 [–4.7 to 0.1] (0.06)	–0.3 [–1.2 to 0.7] (0.582)	–0.3 [–1.2 to 0.5] (0.433)
Race and ethnicity			
Hispanic	–0.6 [–2.8 to 1.7] (0.617)	0.7 [–0.9 to 2.3] (0.405)	0.1 [–0.8 to 1.1] (0.769)
Non-Hispanic Black^b^	2.0 [–0.5 to 4.5] (0.11)	–0.1 [–1.5 to 1.3] (0.89)	0.4 [–0.8 to 1.6] (0.532)
Non-Hispanic White	0.7 [0 to 1.3] (0.048)	0.4 [–0.4 to 1.2] (0.357)	0.3 [0 to 0.6] (0.055)
Sex			
Female	0.8 [–0.2 to 1.7] (0.118)	0.2 [–0.6 to 0.9] (0.664)	0.2 [–0.2 to 0.6] (0.282)
Male	1.4 [0.4 to 2.3] (0.006)	0.8 [0.1 to 1.5] (0.03)	0.4 [–0.2 to 1] (0.18)
Education			
No college	1.4 [0.4 to 2.4] (0.006)	0.4 [–0.9 to 1.6] (0.548)	–0.1 [–0.4 to 0.3] (0.73)
Some college	0.8 [–0.2 to 1.7] (0.102)	0.5 [0 to 1] (0.056)	0.5 [0 to 1] (0.068

^a^ Effects were estimated using the standard difference-in-differences approach described in the methods section. Each outcome was estimated as a function of RCLs, the covariates listed, and state and quarter fixed effects. Standard errors were clustered at the state level and respondents were weighted with standard Behavioral Risk Factor Surveillance System survey weights.

^b^ Lead terms are significant, meaning results should be considered with caution and are not suggestive of the effects of recreational cannabis policy.

### Baseline Sample Characteristics

Prior to RCL implementation, baseline sociodemographic characteristics and alcohol use were not significantly different across states that would and would not implement RCLs. This indicates that respondents in non-RCL states formed a valid comparison with respondents in RCL states. Table 1 reports mean baseline sociodemographic, alcohol use, and cannabis and alcohol policy characteristics across respondents in the treatment and comparison groups (ie, RCL and non-RCL states). We found 2 imbalances. States that implemented RCLs were more likely to have: (1) medical cannabis laws and dispensaries prior to implementing RCLs; and (2) states that implemented RCLs were more likely to have higher tobacco tax rates. This imbalance highlights the importance of robustness checks and limiting the study sample to states that implemented medical cannabis laws during the study period and that had similar tobacco taxes (more details to follow).

### RCLs and Alcohol Use

**Figure 2.**
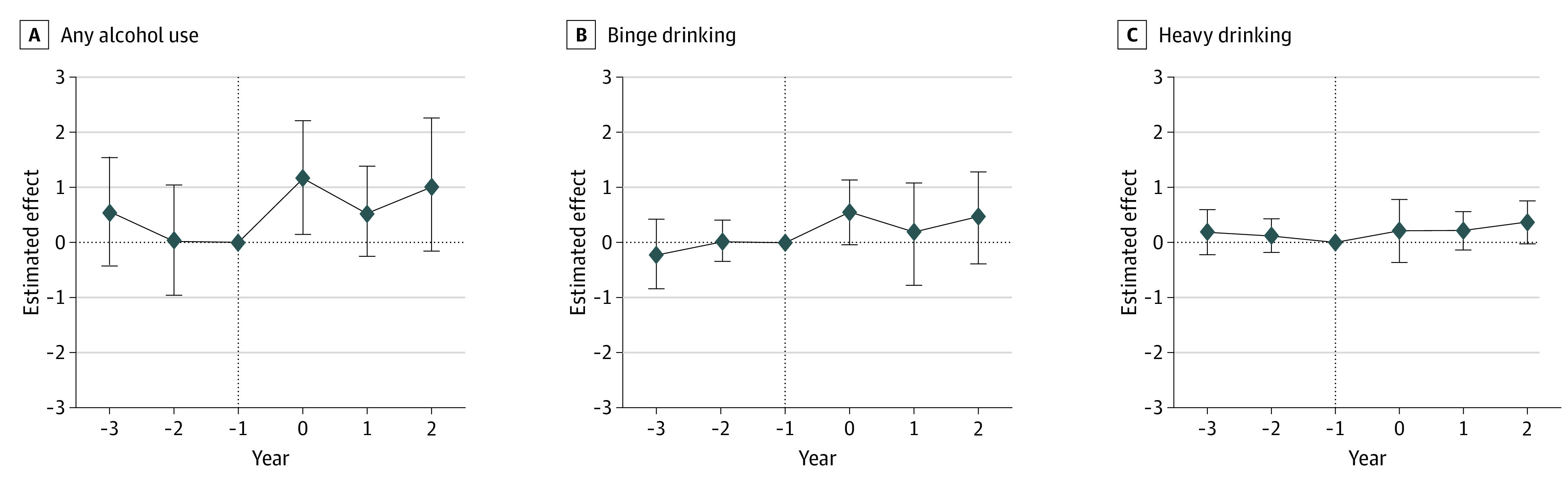
Association of Recreational Cannabis Laws (RCLs) With Drinking Behavior: Event Study Horizontal dotted lines are the point estimates of the difference-in-differences estimates of the association between RCLs and alcohol drinking behavior; vertical dotted lines indicate the Reference; vertical bars represent 95% CIs. Effects were estimated using the standard difference-in-differences approach described in the Methods section. Each outcome was estimated as a function of RCL, the covariates listed, and state and quarter fixed effects. Standard errors were clustered at the state level, and respondents were weighted with standard Behavioral Risk Factor Surveillance System survey weights. Complete results are available in eTable 1 in the Supplement.

We found that RCLs were associated with significant increases in alcohol use that increased in magnitude over time. Table 2 reports the overall pp changes in alcohol use associated with RCLs. Figure 2 reports pp changes in alcohol use over time by year. eTable 1 in the Supplement reports point estimates and confidence intervals for these associations, as well as those for control variables. We found that RCLs were associated with a 0.9 pp increase in any drinking (95% CI, 0.1-1.7; *P* = .02) but were not associated with binge or heavy drinking. The association between RCLs and any drinking was significant and largest in magnitude in the first year of RCL implementation (1.2 pp, 95% CI, 0.1-2.2; *P* = .03). The association was still positive, but not significant in the first and second years after implementation, suggesting that the association between RCLs and any drinking may diminish over time.

### Subgroup Analyses

Subgroup analyses indicated that the study findings were driven by adults aged 18 to 24 years, as well as non-Hispanic White individuals, men, and persons without some college education. Table 2 displays the association of RCLs with alcohol use by age group, race and ethnicity, sex, and education level. The association of RCLs with any drinking among adults aged 18 to 24 was 3.7 pp (95% CI, 1.1-6.3; *P* = .006). This association was not significant among other age groups nor among group-specific associations for binge and heavy drinking. Other subgroups exhibited positive associations between RCLs and any drinking, including non-Hispanic White individuals (0.7 pp; 95% CI, 0.0-1.3; *P* = .048), males (1.4 pp; 95% CI, 0.4-2.3; *P* = .006), and persons without some college education (1.4 pp; 95% CI, 0.4-2.4; *P* = .006). We also found RCLs were associated with a 0.8 pp (95% CI, 0.1 to 1.5; *P* = .03) increase in binge drinking among men, although the association diminished over time.

Although the study’s overall findings suggest that the association between RCLs and any drinking may diminish over time, this was not always observed in the demographic subgroups. As shown in eTable 2 in the Supplement, the association between RCLs and any drinking was persistent over time for adults aged 18 to 24 years, men, and for the first 2 years among persons without some college education. We did not detect a significant association between RCLs and any drinking in any single year for non-Hispanic White individuals. The positive association we found between RCLs and binge drinking among men was only significant during the year of implementation, and it decreased in magnitude to 0.5 pp 2 years after implementation.

### Robustness Checks

The findings were robust to a battery of robustness checks. First, the validity of our DiD analyses was supported by pre-trends checks. With some exceptions—heavy drinking for adults aged 18 to 24, non-Hispanic Black individuals, and women; any drinking for adults aged 65-79 years and persons with no college education—we did not find evidence of differential pre-trends in alcohol use prior to RCL implementation (eTable 2 in the Supplement). Results for these groups should be interpreted with caution. The validity of the DiD analyses also was supported by the balance tests described previously (Table 1). Second, stacked regressions produced similar estimates, indicating that our estimates were robust to bias from the staggered timing of RCLs (eTable 3 in the Supplement). Third, we obtained similar results when we limited the sample to respondents in states that would implement medical cannabis laws during the study period, indicating that the results were robust to imbalances in the treatment and comparison groups with regard to medical cannabis policy (eTable 4 in the Supplement). Fourth, we obtained similar results when limiting comparison states to those that had similar tobacco taxes to treatment states (ie, ≥$2 per pack of cigarettes; eTable 5 in the Supplement). Fifth, we obtained similar results when we conducted the analysis without demographic and policy controls (eTable 6 in the Supplement).

## Discussion

We used a DiD analysis and the nationally representative BRFSS data to estimate the association between RCLs and alcohol use from 2010 through 2019. We found that RCLs were associated with a 0.9 pp increase in any drinking among adults. This increase was driven primarily by adults aged 18 to 24 years, as well as non-Hispanic White individuals, men, and persons without some college education. We also found that RCLs were associated with increased binge drinking among men, although this relationship appeared to dissipate over time. Overall, these findings suggest that increased alcohol use among young adults and men may be an unintended consequence of recreational cannabis legalization.

The first key finding of this analysis is that RCLs were associated with an increase in alcohol use. This result indicates that, at least in the context of RCLs, cannabis and alcohol are economic complements. Our findings are consistent with 2 prior RCL studies that used smaller data from the National Survey on Drug Use and Health,^29^ as well as studies evaluating the association between RCLs and other alcohol-related outcomes, such as alcohol sales^40^ and hospital admissions for alcohol-related diagnoses.^41^ These findings also are consistent with some studies on medical cannabis laws that found that the laws were associated with increased alcohol use^26,27^; however, we acknowledge that recreational and medical cannabis laws differ in terms of the populations that they affect.^38,40^

Our second key finding is that the overall increase in alcohol use was driven by younger adults (18-24 years). We did not find an association between RCLs and alcohol use for older age cohorts. This finding is consistent with those of a recent study which found that younger adults increased cannabis use in response to RCLs.^16^ This finding also is consistent with prior studies on alcohol and cannabis use among college students.^21,22^ However, we found a positive association between alcohol use and RCLs only among persons *without* any college education, suggesting that the positive association between alcohol use and RCLs may not be specifically associated with college environments. We also note that, although we found an overall association between alcohol use and RCLs during the 3 years following RCL implementation, the event-study results suggest that this relationship may diminish over time. Future studies should determine how long the association between alcohol use and RCLs persists after implementation or diminishes along with a perceived novelty of recreational cannabis. Nevertheless, these results suggest that increased alcohol use owing to recreational cannabis is primarily a concern among younger adults.

The last key finding is that increases in alcohol use were also substantial among men. We found stronger increases in any alcohol use among men, a trend which persisted over time. We also found a significant association between binge drinking and RCLs among men, although it may have diminished over time. These findings are consistent with the literature on cannabis laws and alcohol use—which find that alcohol consumption among men is more responsive to cannabis liberalization policies than it is among women—indicating that newer RCLs affect the population in much the same way as older RCLs and medical cannabis laws did.^26,42,43^

Exploring the association of cannabis legalization and alcohol use is especially important given the rapid pace at which states are passing RCLs. Cannabis legalization increases cannabis use,^15^ which by itself has many health-related costs (eg, cannabis use disorder, lung damage) and benefits (eg, treatment of chronic pain, glaucoma, epilepsy).^17^ However, cannabis use also has costs and benefits regarding substances that people may use more of or less of when using cannabis (ie, complements and substitutes). An expanding literature suggests, albeit not conclusively, that cannabis legalization may reduce opioid use and associated risks.^44,45^ However, our findings suggest that these gains may need to be considered against the increased costs of alcohol use among young adults and men. As more data from the postlegalization period becomes available, cost-benefit analyses would be immensely informative to the debate surrounding cannabis legalization, as would additional studies on the relationship between RCLs and alcohol use.

### Limitations

This study had some limitations. First, the BRFSS did not measure cannabis use in many states because states are not required to include an optional question for states to include in their survey.^46^ However, prior literature finds that RCLs increased cannabis use during our study period.^47,48^ Second, BRFSS respondents may underestimate alcohol use owing to underreporting and non-response bias.^49,50^ This measurement error is only problematic for our DiD approach if it is systematically related to which states implemented RCLs, which is unlikely. Third, RCLs may have different effects across states owing to states’ particular approaches to cannabis legalization, as well as cultural perceptions of cannabis use. Therefore, our estimates represent the average association between RCLs and alcohol use in states that implemented these laws.

## Conclusions

In this cross-sectional study with a DiD analysis, we found that RCLs were associated with increased alcohol use, particularly among younger adults. This finding highlights one of several important trade-offs implicit in cannabis liberalization. Cannabis use also poses its own health risks, yet its legalization has been associated with decreases in opioid prescribing, temporary reductions in opioid-related emergency department visits, and does not appear to increase the use of other substances. Policy makers and clinicians should consider changes in substance use patterns as states continue to legalize recreational cannabis. Future studies should continue to examine the relationship between alcohol and cannabis use, both over longer periods of time and in terms of other health outcomes.

## Notes

Supplement. **eTable 1. **Associations of Recreational Cannabis Laws and Sociodemographic and Policy Controls with Drinking Behavior **eTable 2.** Associations of Recreational Cannabis Laws with Drinking Behavior: F-Statistic for Test of Joint Significance of Leads Prior to Implementation, Individual Leads Prior to Implementation, and Lags After Implementation, Overall and by Sociodemographic Groups **eTable 3.** Associations of Recreational Cannabis Laws with Drinking Behavior: Stacked Difference-in-Differences Estimates, Overall and by Demographic Groups **eTable 4.** Association of Recreational Cannabis Laws with Drinking Behavior: Difference-in-Differences Estimates with Sample Limited to States that Implemented a Medical Cannabis Law, Overall and by Sociodemographic Groups **eTable 5. **Association of Recreational Cannabis Laws with Drinking Behavior: Difference-in-Differences Estimates without Demographic and Policy Covariates, Overall and by Sociodemographic Groups **eTable 6.** Association of Recreational Cannabis Laws with Drinking Behavior: Difference-in-Differences Estimates without Demographic and Policy Covariates, Overall and by Sociodemographic Groups
