# Supplementary material for: Association of Recreational Cannabis Legalization With Alcohol Use Among Adults in the US, 2010 to 2019
Source: JAMA Health Forum. 2022 Nov 18;3(11):e224069. doi: 10.1001/jamahealthforum.2022.4069 (PMC9675003; doi:10.1001/jamahealthforum.2022.4069)
Supplement: Supplement. — eTable 1. Associations of Recreational Cannabis Laws and Sociodemographic and Policy Controls with Drinking Behavior eTable 2. Associations of Recreational Cannabis Laws with Drinking Behavior: F-Statistic for Test of Joint Significance of Leads Prior to Implementation, Individual Leads Prior to Implementation, and Lags After Implementation, Overall and by Sociodemographic Groups eTable 3. Associations of Recreational Cannabis Laws with Drinking Behavior: Stacked Difference-in-Differences Estimates, Overall and by Demographic Groups eTable 4. Association of Recreational Cannabis Laws with Drinking Behavior: Difference-in-Differences Estimates with Sample Limited to States that Implemented a Medical Cannabis Law, Overall and by Sociodemographic Groups eTable 5. Association of Recreational Cannabis Laws with Drinking Behavior: Difference-in-Differences Estimates without Demographic and Policy Covariates, Overall and by Sociodemographic Groups eTable 6. Association of Recreational Cannabis Laws with Drinking Behavior: Difference-in-Differences Estimates without Demographic and Policy Covariates, Overall and by Sociodemographic Groups [file jamahealthforum-e224069-s001.pdf]

# Supplemental Online Content

Macha V, Abouk R, Drake C. Association of recreational cannabis legalization with alcohol use among adults in the US, 2010 to 2019. *JAMA Health Forum.* 2022:3(11):e224069. doi:10.1001/jamahealthforum.2022.4069

**eTable 1.** Associations of Recreational Cannabis Laws and Sociodemographic and Policy Controls with Drinking Behavior

**eTable 2.** Associations of Recreational Cannabis Laws with Drinking Behavior: F-Statistic for Test of Joint Significance of Leads Prior to Implementation, Individual Leads Prior to Implementation, and Lags After Implementation, Overall and by Sociodemographic Groups

**eTable 3.** Associations of Recreational Cannabis Laws with Drinking Behavior: Stacked Difference-in-Differences Estimates, Overall and by Demographic Groups

**eTable 4.** Association of Recreational Cannabis Laws with Drinking Behavior: Difference-in-Differences Estimates with Sample Limited to States that Implemented a Medical Cannabis Law, Overall and by Sociodemographic Groups

**eTable 5.** Association of Recreational Cannabis Laws with Drinking Behavior: Difference-in-Differences Estimates without Demographic and Policy Covariates, Overall and by Sociodemographic Groups

**eTable 6.** Association of Recreational Cannabis Laws with Drinking Behavior: Difference-in-Differences Estimates without Demographic and Policy Covariates, Overall and by Sociodemographic Groups

This supplemental material has been provided by the authors to give readers additional information about their work.

© 2022 Macha V et al. *JAMA Health Forum.*

**eTable 1. Associations of Recreational Cannabis Laws and Sociodemographic and Policy Controls with Drinking Behavior**

| Covariate | Drinking Behavior (Percentage Point Association, 95% CI, *P* Value) | | |
|---|---|---|---|
| | **Any Drinking** | **Binge Drinking** | **Heavy Drinking** |
| Year Relative to Recreational Cannabis Law Implementation | | | |
| 3 Years Prior | 0.55 [-0.43, 1.53] 0.265 | -0.22 [-0.85, 0.41] 0.495 | 0.19 [-0.22, 0.6] 0.357 |
| 2 Years Prior | 0.04 [-0.96, 1.03] 0.939 | 0.03 [-0.34, 0.4] 0.876 | 0.12 [-0.18, 0.43] 0.41 |
| 1 Year Prior | - | - | - |
| Implementation Year | 1.16 [0.14, 2.19] 0.026 | 0.54 [-0.04, 1.13] 0.066 | 0.21 [-0.36, 0.78] 0.46 |
| 1 Year Post | 0.56 [-0.26, 1.38] 0.177 | 0.16 [-0.77, 1.08] 0.736 | 0.21 [-0.13, 0.56] 0.217 |
| 2 Years Post | 1.05 [-0.16, 2.26] 0.089 | 0.45 [-0.38, 1.27] 0.283 | 0.37 [-0.02, 0.76] 0.063 |
| Age | | | |
| 18-24 | - | - | - |
| 25-34 | -4.87 [-15.95, 6.2] 0.381 | 0.64 [-9.28, 10.55] 0.898 | -0.86 [-5.36, 3.64] 0.702 |
| 35-49 | -25.34 [-40.69, -10] 0.002 | -17.04 [-26.39, -7.7] 0.001 | -6.12 [-10.11, -2.12] 0.003 |
| 50-64 | -21.53 [-33.56, -9.5] 0.001 | -23.56 [-33.22, -13.9] 0 | -6.91 [-12.49, -1.33] 0.016 |
| 65-79 | -17.88 [-35.23, -0.53] 0.044 | -19.7 [-31.33, -8.07] 0.001 | -8.3 [-14.66, -1.95] 0.011 |
| 80+ | -26.18 [-50.59, -1.77] 0.036 | -24.58 [-43.63, -5.53] 0.012 | -1.14 [-10.93, 8.64] 0.815 |
| Missing | -35.97 [-77.83, 5.88] 0.09 | -16.75 [-40.83, 7.33] 0.169 | -7.17 [-27.5, 13.15] 0.482 |
| Race/Ethnicity | | | |
| Non-Hispanic White | - | - | - |
| Non-Hispanic Black | 2.07 [-2.67, 6.81] 0.384 | -1.66 [-4.55, 1.24] 0.256 | -0.53 [-2.6, 1.53] 0.607 |
| Hispanic | -0.52 [-4.31, 3.26] 0.783 | 0.65 [-1.44, 2.73] 0.536 | -1.18 [-2.65, 0.3] 0.115 |
| Other | -5.39 [-8.93, -1.86] 0.004 | -4.95 [-7.39, -2.51] 0 | -3.29 [-5.14, -1.44] 0.001 |
| Binary Covariates | | | |
| Female | -8.22 [-14.29, -2.15] 0.009 | -9.74 [-15.19, -4.29] 0.001 | -2.63 [-5.62, 0.36] 0.083 |
| Some College[b] | 24.33 [18.62, 30.04] 0 | 3.48 [-1.55, 8.51] 0.171 | -0.09 [-4.11, 3.92] 0.963 |
| Student | -18.74 [-34.75, -2.74] 0.023 | -15.67 [-25.45, -5.88] 0.002 | -2.53 [-8.41, 3.35] 0.391 |
| Unemployed | 5.45 [-7.16, 18.07] 0.389 | -0.65 [-8.53, 7.23] 0.869 | 0.05 [-5.64, 5.75] 0.985 |
| Has Child | 6.1 [-4.32, 16.51] 0.245 | 0.25 [-4.66, 5.17] 0.918 | -1.1 [-4.22, 2.02] 0.482 |
| Married | 3.97 [-3.6, 11.54] 0.297 | 1.09 [-5.82, 8.01] 0.752 | 3.44 [-0.57, 7.45] 0.091 |
| Income over $50,000 | 30.18 [20.64, 39.73] 0 | 7.72 [2.71, 12.73] 0.003 | 3.83 [0.97, 6.7] 0.01 |
| Policy Covariates | | | |
| Medical Cannabis Law | -0.7 [-1.22, -0.18] 0.01 | -0.47 [-0.89, -0.05] 0.028 | -0.08 [-0.27, 0.1] 0.353 |
| Medical Dispensary | -0.44 [-0.95, 0.07] 0.089 | -0.33 [-0.74, 0.07] 0.106 | -0.04 [-0.18, 0.1] 0.561 |
| Beer Tax Rate | 2.08 [-0.85, 5.01] 0.16 | 0.64 [-0.7, 1.97] 0.342 | 0.48 [-0.31, 1.26] 0.232 |
| Cigarette Tax Rate | 0.09 [-0.24, 0.43] 0.575 | 0.05 [-0.17, 0.27] 0.653 | -0.02 [-0.18, 0.15] 0.853 |
| Behavioral Risk Factor Surveillance System Response Location | | | |
| Non-MSA[c] | - | - | - |
| MSA[c] | -7.36 [-12.55, -2.17] 0.006 | -4.29 [-6.99, -1.59] 0.002 | -2.2 [-3.44, -0.96] 0.001 |
| Cell Phone | 2.48 [-0.16, 5.11] 0.065 | 1.17 [-0.81, 3.15] 0.24 | 1.04 [-0.18, 2.26] 0.093 |

© 2022 Macha V et al. *JAMA Health Forum.*

[a] Effects are estimated using a standard differences-in-differences approach described in the methods section. Each outcome is estimated as a function of recreational cannabis legalization, the covariates listed above, and state and quarter fixed effects. Standard errors were clustered at the state level and respondents were weighted with standard Behavioral Risk Factor Surveillance System survey weights.

[b] Refers to respondents that have received some college or technical school education or graduated.

[c] Abbreviation: Metropolitan statistical area

© 2022 Macha V et al. *JAMA Health Forum.*

**eTable 2. Associations of Recreational Cannabis Laws with Drinking Behavior: F-Statistic for Test of Joint Significance of Leads Prior to Implementation, Individual Leads Prior to Implementation, and Lags After Implementation, Overall and by Sociodemographic Groups[a]**

| Sociodemographic Group, Drinking Behavior | Effect of Recreational Cannabis Law on Drinking Behavior (Percentage Points) | | | | | | |
|---|---|---|---|---|---|---|---|
| | | Year Relative to Recreational Cannabis Law Implementation | | | | | |
| | F-Statistic | -3 | -2 | -1 | 0 | 1 | 2 |
| **Overall** | | | | | | | |
| Any | 0.3 | 0.5 | 0 | - | 1.2* | 0.6 | 1 |
| Binge | -0.1 | -0.2 | 0 | - | 0.5 | 0.2 | 0.4 |
| Heavy | 0.1 | 0.2 | 0.1 | - | 0.2 | 0.2 | 0.4 |
| **Age: 18-24** | | | | | | | |
| Any | 0.6 | 1.6 | -0.4 | - | 3.8* | 2.7* | 4.5* |
| Binge | 0.2 | 0.4 | 0.1 | - | 2 | -0.1 | 1.3 |
| Heavy[b] | 0.9* | 1.3 | 0.6 | - | 0.8 | -0.9 | 0.2 |
| **Age: 25-34** | | | | | | | |
| Any | 1.5 | 1.6 | 1.5 | - | 2 | 0.6 | 0.5 |
| Binge | 0.2 | -1.1 | 1.3 | - | -0.8 | 0.7 | 0.2 |
| Heavy | 0.6 | 0.4 | 0.7 | - | 0.5 | 0.9 | 1* |
| **Age: 35-49** | | | | | | | |
| Any | 0.1 | 0.9 | -0.7 | - | 1 | 1.6 | 0.5 |
| Binge | 0.2 | 0.2 | -0.3 | - | 0.2 | 1.3* | 0.7 |
| Heavy | 0.2 | 0.4 | 0.1 | - | -0.1 | 0.5 | 0.4 |
| **Age: 50-64** | | | | | | | |
| Any | -0.5 | -0.7 | -0.4 | - | 0.3 | -0.1 | 0.6 |
| Binge | 0.2 | -0.1 | 0 | - | 1.6* | 0.1 | 0.1 |
| Heavy | -0.2 | -0.3 | 0 | - | 0.3 | 0.1 | 0.6 |
| **Age: 65-79** | | | | | | | |
| Any[a] | 1.1* | 1.5* | 0.8 | - | 1.2 | 1 | 0.7 |
| Binge | 0.2 | 0.6 | 0.1 | - | 0.1 | -1.2* | 0.6 |
| Heavy | 0.1 | 0.2 | 0 | - | 0.1 | 0 | -0.3 |
| **Age: 80+** | | | | | | | |
| Any[a] | -2.1 | -3.2 | -0.9 | - | -2.7 | -4.4* | 0.7 |
| Binge | 0.2 | -0.5* | -0.8 | - | -0.4 | -0.6 | 0.2 |
| Heavy | -0.7 | -0.2 | -1.2 | - | -0.4 | -0.3 | -0.3 |
| **Race/Ethnicity: Non-Hispanic White** | | | | | | | |
| Any | -0.2 | -0.2 | -0.3 | - | 0.8 | 0.6 | 0.6 |
| Binge | -0.3 | -0.5 | -0.1 | - | 0.3 | 0.3 | 0.5 |
| Heavy | -0.1 | -0.1 | 0 | - | 0.2 | 0.3 | 0.4* |
| **Race/Ethnicity: Non-Hispanic Black** | | | | | | | |
| Any | 3.1* | 4* | 2.4* | - | 2.6* | 0.4 | 2.7 |
| Binge | -0.3 | 1 | 2.2* | - | 0 | 0.1 | -0.3 |
| Heavy | 0.1 | -0.1 | 0.4 | - | 0 | -0.1 | 1.6 |
| **Race/Ethnicity: Hispanic** | | | | | | | |
| Any | -1.4 | -1.6 | -1.3 | - | -0.1 | -0.6 | -1 |
| Binge | -0.3 | -2* | -0.1 | - | 1.5 | 0.1 | 0.6 |
| Heavy | 0.2 | 0 | 0.3 | - | 0.3 | 0.1 | 0 |
| **Sex: Male** | | | | | | | |
| Any | 0.1 | 0.2 | 0 | - | 1.1* | 1.4* | 1.7* |
| Binge | -0.1 | -0.4 | 0.1 | - | 0.9* | 0.9 | 0.5 |
| Heavy | 0.1 | 0.3 | -0.1 | - | 0.2 | 0.5 | 0.6 |
| **Sex: Female** | | | | | | | |

© 2022 Macha V et al. *JAMA Health Forum.*

| | | | | | | | |
|---|---|---|---|---|---|---|---|
| Any | 0.7* | 1.1* | 0.4 | - | 1.4 | 0.1 | 0.5 |
| Binge | -0.1 | 0.2 | 0.3 | - | 0.3 | -0.3 | 0.5 |
| Heavy | 0.4 | 0.3 | 0.5* | - | 0.3 | 0.1 | 0.2 |
| **Education: No College** | | | | | | | |
| Any | -0.2 | 0.3 | -0.7 | - | 1.6* | 1.4* | 1.1 |
| Binge | -0.2 | -0.2 | -0.2 | - | 0.6 | 0 | 0.5 |
| Heavy | 0.4* | 0.4 | 0.4* | - | 0 | -0.3 | 0.1 |
| **Education: Some College** | | | | | | | |
| Any | 0.7 | 0.7 | 0.6 | - | 1 | 0.3 | 1.1 |
| Binge | -0.2 | -0.1 | 0.4* | - | 0.7* | 0.4 | 0.5 |
| Heavy | 0.1 | 0.1 | 0 | - | 0.4 | 0.5* | 0.5 |

[a] Effects are estimated using a standard differences-in-differences approach described in the methods section. Each outcome is estimated as a function of recreational cannabis legalization, demographic and policy controls, and state-stack and quarter-stack fixed effects. Standard errors were clustered at the state level and respondents were weighted with standard Behavioral Risk Factor Surveillance System survey weights. The F-Statistic reports the results of a test on the joint significance of the lead terms.

[b] Lead terms are significant, meaning results should be considered with caution and not suggestive of the effects of recreational cannabis policy.

* $p < 0.05$ ** $p < 0.01$ *** $p < 0.001$

© 2022 Macha V et al. *JAMA Health Forum.*

**eTable 3. Associations of Recreational Cannabis Laws with Drinking Behavior: Stacked Difference-in-Differences Estimates, Overall and by Demographic Groups[a]**

| Sociodemographic Group | Effect of Recreational Cannabis Law on Drinking Behavior (Percentage Point Association, 95% CI, *P* Value) | | |
|---|---|---|---|
| | **Any Drinking** | **Binge Drinking** | **Heavy Drinking** |
| **Overall** | **1 [0.1, 2] 0.039** | 0.4 [-0.1, 1] 0.14 | 0.3 [-0.2, 0.7] 0.239 |
| **Age** | | | |
| 18-24 | **3.2 [0.9, 5.5] 0.006** | 1.3 [0, 2.7] 0.057 | -0.4 [-1.3, 0.5] 0.35 |
| 25-34 | 0.8 [-0.9, 2.4] 0.34 | -0.2 [-1.7, 1.3] 0.767 | 0.6 [-0.2, 1.5] 0.156 |
| 35-49 | 1 [-0.8, 2.7] 0.271 | 0.7 [-0.2, 1.5] 0.122 | 0.1 [-0.3, 0.6] 0.545 |
| 50-64 | 0.1 [-0.9, 1.1] 0.851 | 0.4 [-0.4, 1.3] 0.313 | 0.4 [-0.3, 1] 0.243 |
| 65-79 | **1.5 [0.5, 2.5] 0.003** | -0.1 [-0.6, 0.4] 0.692 | 0.1 [-0.7, 0.8] 0.822 |
| 80+ | -1.4 [-3.4, 0.6] 0.159 | -0.4 [-1.4, 0.7] 0.488 | -0.1 [-0.6, 0.5] 0.818 |
| **Race/Ethnicity** | | | |
| Non-Hispanic White | 0.4 [-0.2, 1] 0.166 | 0.3 [-0.4, 1] 0.341 | 0.4 [-0.1, 0.8] 0.129 |
| Non-Hispanic Black | **2.1 [-0.3, 4.4] 0.082** | -0.1 [-1.2, 1.1] 0.88 | 0.4 [-0.8, 1.6] 0.485 |
| Hispanic | -0.2 [-2.1, 1.8] 0.859 | 0.3 [-0.7, 1.2] 0.574 | -0.2 [-0.7, 0.3] 0.401 |
| **Gender** | | | |
| Male | **1.3 [0.4, 2.2] 0.007** | **0.9 [0.2, 1.6] 0.014** | 0.4 [-0.2, 0.9] 0.22 |
| Female | 0.9 [-0.3, 2.1] 0.143 | 0 [-0.6, 0.5] 0.875 | 0.2 [-0.2, 0.6] 0.332 |
| **Education** | | | |
| No College | **1.3 [0.6, 2.1] 0.001** | 0.5 [-0.6, 1.6] 0.391 | -0.1 [-0.4, 0.3] 0.662 |
| Some College | 0.9 [-0.3, 2.2] 0.15 | 0.5 [0, 1] 0.051 | 0.5 [-0.1, 1.1] 0.093 |

[a] Effects are estimated using a stacked differences-in-differences approach as in Cengiz et al. (2019). This approach eliminates comparisons between states that implemented recreational cannabis laws later to those that implement recreational cannabis laws earlier that can add bias to standard difference-in-differences estimates. Each outcome is estimated as a function of recreational cannabis legalization, demographic and policy controls, and state-stack and quarter-stack fixed effects. Standard errors were clustered at the state level and respondents were weighted with standard Behavioral Risk Factor Surveillance System survey weights.

© 2022 Macha V et al. *JAMA Health Forum.*

**eTable 4. Association of Recreational Cannabis Laws with Drinking Behavior: Difference-in-Differences Estimates with Sample Limited to States that Implemented a Medical Cannabis Law, Overall and by Sociodemographic Groups**

| Sociodemographic Group | Effect of Recreational Cannabis Law on Drinking Behavior (Percentage Point Association, 95% CI, *P* Value) | | |
|---|---|---|---|
| | **Any Drinking** | **Binge Drinking** | **Heavy Drinking** |
| **Overall** | **0.8 [0.1, 1.6] 0.03** | 0.4 [-0.2, 1.1] 0.207 | 0.1 [-0.2, 0.4] 0.445 |
| **Age** | | | |
| 18-24 | **3.3 [0, 6.5] 0.048** | 1.2 [-0.5, 2.8] 0.165 | 0.2 [-1, 1.5] 0.71 |
| 25-34 | 1.4 [-0.3, 3.1] 0.1 | -0.3 [-1.9, 1.3] 0.704 | 0.4 [-0.5, 1.3] 0.339 |
| 35-49 | 1 [-0.6, 2.7] 0.223 | 1.1 [0, 2.1] 0.053 | 0 [-0.6, 0.6] 0.974 |
| 50-64 | 0.3 [-1.1, 1.6] 0.683 | 0.7 [-0.5, 1.9] 0.224 | 0.2 [-0.7, 1] 0.661 |
| 65-79 | 0.7 [-0.6, 2] 0.278 | -0.1 [-0.6, 0.4] 0.583 | 0 [-0.7, 0.8] 0.988 |
| 80+ | -2.6 [-5.3, 0] 0.052 | -0.3 [-1.4, 0.8] 0.6 | -0.6 [-1.5, 0.4] 0.252 |
| **Race/Ethnicity** | | | |
| Non-Hispanic White | **0.7 [0.1, 1.3] 0.03** | 0.4 [-0.4, 1.2] 0.289 | 0.1 [-0.2, 0.5] 0.434 |
| Non-Hispanic Black | 1.4 [-0.9, 3.8] 0.227 | -0.1 [-1.4, 1.2] 0.873 | 0.4 [-0.6, 1.4] 0.415 |
| Hispanic | 0 [-2.2, 2.3] 0.98 | 0.4 [-1.3, 2.1] 0.631 | -0.1 [-1, 0.8] 0.815 |
| **Gender** | | | |
| Male | **1 [0.2, 1.9] 0.019** | **0.7 [0, 1.4] 0.037** | 0.2 [-0.3, 0.7] 0.444 |
| Female | 0.8 [-0.2, 1.7] 0.109 | 0.2 [-0.6, 1] 0.562 | 0.1 [-0.3, 0.4] 0.612 |
| **Education** | | | |
| No College | **1.3 [0.1, 2.4] 0.032** | 0.3 [-1, 1.6] 0.65 | -0.1 [-0.6, 0.3] 0.497 |
| Some College | 0.7 [-0.2, 1.6] 0.111 | **0.6 [0.1, 1.1] 0.024** | 0.2 [-0.1, 0.6] 0.168 |

[a] Effects were estimated limiting the sample to persons in states that implemented a medical cannabis law during the study period. This approach may provide a more similar comparison group for persons in states that implemented recreational cannabis laws. Each outcome was estimated as a function of recreational cannabis legalization, demographic and policy controls, and state and quarter fixed effects. Standard errors were clustered at the state level and respondents were weighted with standard Behavioral Risk Factor Surveillance System survey weights.

**eTable 5. Association of Recreational Cannabis Laws with Drinking Behavior: Difference-in-Differences Estimates with Sample Limited to Treatment States and Comparison States with Similar Tobacco Taxes, Overall and by Sociodemographic Groups**

| Sociodemographic Group | Effect of Recreational Cannabis Law on Drinking Behavior (Percentage Point Association, 95% CI, *P* Value) | | |
|---|---|---|---|
| | **Any Drinking** | **Binge Drinking** | **Heavy Drinking** |
| **Overall** | 0.7 [-0.1, 1.5] 0.083 | 0.4 [-0.2, 1.1] 0.191 | 0.2 [-0.2, 0.6] 0.302 |
| **Age** | | | |
| 18-24 | 3.4 [0.3, 6.4] 0.032 | 1.4 [-0.3, 3.1] 0.107 | 0 [-1.4, 1.4] 0.984 |
| 25-34 | 1.1 [-0.9, 3] 0.269 | 0.1 [-1.8, 1.9] 0.934 | 0.8 [-0.3, 1.9] 0.154 |
| 35-49 | 0.8 [-0.9, 2.5] 0.34 | 0.9 [-0.1, 2] 0.074 | 0.2 [-0.4, 0.8] 0.589 |
| 50-64 | 0 [-1.3, 1.3] 0.972 | 0.6 [-0.5, 1.7] 0.299 | 0.2 [-0.6, 1] 0.637 |
| 65-79 | 0.9 [-0.5, 2.3] 0.181 | -0.2 [-0.7, 0.4] 0.536 | 0 [-0.7, 0.7] 0.977 |
| 80+ | -2.2 [-4.6, 0.2] 0.066 | -0.1 [-1.1, 0.8] 0.778 | -0.4 [-1.5, 0.6] 0.39 |
| **Race/Ethnicity** | | | |
| Non-Hispanic White | 0.6 [0, 1.2] 0.042 | 0.5 [-0.3, 1.2] 0.228 | 0.2 [-0.2, 0.6] 0.224 |
| Non-Hispanic Black | 1.7 [-0.8, 4.2] 0.174 | 0 [-1.5, 1.5] 0.984 | 0.5 [-0.7, 1.7] 0.402 |
| Hispanic | -1 [-3.5, 1.4] 0.386 | 0.5 [-1.1, 2.1] 0.536 | 0 [-0.9, 0.9] 0.961 |
| **Gender** | | | |
| Male | 1.3 [0.3, 2.3] 0.011 | 1 [0.2, 1.8] 0.012 | 0.4 [-0.3, 1.1] 0.227 |
| Female | 0.6 [-0.6, 1.7] 0.316 | 0.3 [-0.5, 1.1] 0.465 | 0.1 [-0.3, 0.5] 0.501 |
| **Education** | | | |
| No College | 1.1 [-0.1, 2.3] 0.062 | 0.3 [-1.1, 1.7] 0.657 | -0.1 [-0.6, 0.3] 0.48 |
| Some College | 0.6 [-0.3, 1.5] 0.199 | 0.7 [0.1, 1.2] 0.017 | 0.4 [-0.1, 1] 0.099 |

[a] Effects were estimated limiting the sample to persons in states that (a) implemented a recreational cannabis law during the study period, or (b) had at least a two dollar tobacco tax per pack of cigarettes. This approach provides a more similar comparison group in terms of tobacco taxes. Specifically, mean tobacco taxes per pack were $3.12 in the treatment group. In the baseline comparison group, they were $2.50. In the comparison group used here, they are $3.05. Each outcome was estimated as a function of recreational cannabis legalization, demographic and policy controls, and state and quarter fixed effects. Standard errors were clustered at the state level and respondents were weighted with standard Behavioral Risk Factor Surveillance System survey weights.

© 2022 Macha V et al. *JAMA Health Forum.*

**eTable 6. Association of Recreational Cannabis Laws with Drinking Behavior: Difference-in-Differences Estimates without Demographic and Policy Covariates, Overall and by Sociodemographic Groups**

| Sociodemographic Group | Effect of Recreational Cannabis Law on Drinking Behavior (Percentage Point Association, 95% CI, *P* Value) | | |
|---|---|---|---|
| | **Any Drinking** | **Binge Drinking** | **Heavy Drinking** |
| **Overall** | **0.9 [0.1, 1.7] 0.021** | 0.4 [-0.3, 1] 0.241 | 0.3 [-0.1, 0.6] 0.171 |
| **Age** | | | |
| 18-24 | **3.7 [1.1, 6.3] 0.006** | 1.1 [-0.5, 2.8] 0.179 | 0.1 [-1.2, 1.4] 0.883 |
| 25-34 | 1.1 [-0.7, 3] 0.226 | -0.1 [-1.7, 1.6] 0.951 | 0.8 [-0.2, 1.7] 0.105 |
| 35-49 | 1.1 [-0.7, 2.8] 0.239 | 0.7 [-0.2, 1.6] 0.125 | 0.2 [-0.3, 0.8] 0.398 |
| 50-64 | 0.3 [-1.1, 1.6] 0.674 | 0.7 [-0.3, 1.8] 0.177 | 0.3 [-0.5, 1.1] 0.419 |
| 65-79 | 1 [-0.2, 2.2] 0.104 | -0.2 [-0.7, 0.3] 0.443 | 0 [-0.7, 0.6] 0.911 |
| 80+ | -2.3 [-4.7, 0.1] 0.06 | -0.3 [-1.2, 0.7] 0.582 | -0.3 [-1.2, 0.5] 0.433 |
| **Race/Ethnicity** | | | |
| Non-Hispanic White | **0.7 [0, 1.3] 0.048** | 0.4 [-0.4, 1.2] 0.357 | **0.3 [0, 0.6] 0.055** |
| Non-Hispanic Black | 2 [-0.5, 4.5] 0.11 | -0.1 [-1.5, 1.3] 0.89 | 0.4 [-0.8, 1.6] 0.532 |
| Hispanic | -0.6 [-2.8, 1.7] 0.617 | 0.7 [-0.9, 2.3] 0.405 | 0.1 [-0.8, 1.1] 0.769 |
| **Gender** | | | |
| Male | **1.4 [0.4, 2.3] 0.006** | **0.8 [0.1, 1.5] 0.031** | 0.4 [-0.2, 1] 0.18 |
| Female | 0.8 [-0.2, 1.7] 0.118 | 0.2 [-0.6, 0.9] 0.664 | 0.2 [-0.2, 0.6] 0.282 |
| **Education** | | | |
| No College | **1.4 [0.4, 2.4] 0.006** | 0.4 [-0.9, 1.6] 0.548 | -0.1 [-0.4, 0.3] 0.73 |
| Some College | 0.8 [-0.2, 1.7] 0.102 | 0.5 [0, 1] 0.056 | 0.5 [0, 1] 0.068 |

[a] Effects were estimated limiting the sample to persons in states that implemented a medical cannabis law during the study period. This approach may provide a more similar comparison group for persons in states that implemented recreational cannabis laws. Each outcome was estimated as a function of recreational cannabis legalization, demographic and policy controls, and state and quarter fixed effects. Standard errors were clustered at the state level and respondents were weighted with standard Behavioral Risk Factor Surveillance System survey weights.

© 2022 Macha V et al. *JAMA Health Forum.*
